# Enhancement of various images using coefficients obtained from a class of Sakaguchi type functions

**DOI:** 10.1038/s41598-023-45938-y

**Published:** 2023-10-31

**Authors:** B. Aarthy, B. Srutha Keerthi

**Affiliations:** Division of Mathematics, School of Advanced Sciences, Vellore Institute of Technology, Chennai, 600 127 India

**Keywords:** Engineering, Mathematics and computing

## Abstract

Digital image processing has a wide range of uses, including robotics and automated inspection of industrial parts. Other uses include remote sensing using satellites and other spacecraft, image transmission and storage for business applications, medical processing, and Acoustic image processing. The process of highlighting particular intriguing features in a hidden image is known as image enhancement. We can accomplish this by altering the brightness, contrast, etc. The generated output is more suitable than the original image for some particular purposes. The proposed algorithm which is based on the convolution of coefficient bounds of a subclass $p-\Upsilon {\mathcal {S}}^*(t,\delta ,\mu )$ obtained using Mittag-Leffler type Poisson Distribution is tested on three image data sets with different dimensions and image formats (PNG, JPEG, TIFF, etc.) and its PSNR, SSIM, MSE, RMSE, PCC and MAE values are observed to check the quality of the enhanced images.

## Introduction

The practise of applying different techniques to an image in order to enhance it or extract useful information from it is known as image processing. It is a form of signal processing in which a picture serves as the input, and the output may be another image, features, or characteristics associated with the input image. One of the technologies that is currently developing swiftly is image processing. It is a major area of study in the fields of engineering and computer science. The following three phases fundamentally make up image processing: Importing the image with the aid of image acquisition tools, reviewing and editing the image, and creating a report or modified image as a result of the analysis. Analog and digital image processing are the two categories of image processing techniques. The tangible copies, such as prints and photographs, can be processed using analogue image technology. Applying these visual techniques, image analysts employ several interpretational fundamentals. Through the use of computers, digital image processing techniques enable image alteration. Pre-processing, enhancement, and display, as well as information extraction, are the three general processes that all sorts of data must go through when employing digital approach. Image enhancement is the process of modifying digital images to provide outcomes that are better suited for display or additional image analysis. That is, the original image is processed so that the resultant image is more suitable. To spot important details of the image, the process includes removing noise, sharpening or brightening of the image. The goal of image enhancement is to make it easier for viewers to understand and gain more insight from the information in images. While using this technique, the properties of image are modified. Many techniques are used to enhance a digital image without wrecking the actual image. There are two main categories of image enhancement techniques available:

(i) Spatial Domain method.
(ii) Frequency Domain method.

The image plane, which is made up of all the pixels that make up an image, is referred to as the Spatial domain whereas Frequency Domain methods are based on altering the Fourier Transformation of the image. Our primary method in this article is the Spatial Domain in which a $2\times 2$ matrix with each member representing the intensity of a pixel can be used to represent a grayscale image. The spatial domain method can be represented as:

$$\begin{aligned} v(x,y) = {\mathcal {T}}[u(x,y)] \end{aligned}$$

where *u*(*x*, *y*) is the input image, *v*(*x*, *y*) is the processed image and ${\mathcal {T}}$ is defined to be an operator on *u* over some neighborhood of *u*(*x*, *y*). The operator ${\mathcal {T}}$ can operate on a set of input images. In order to provide an acceptable result, some applications such as convolution are required by the image enhancement task. Convolution is a straightforward mathematical operation that forms the basis for several popular image processing techniques. By “multiplying together” two arrays of numbers, typically of different sizes but of the same dimensionality, convolution offers a method for creating a third array of numbers, also of the same dimensionality. Using this, image processing operators can be implemented whose output pixel values are straightforward linear combinations of certain input pixel values.

In this paper, we use the method of convolution on the coefficients of the class obtained using the Mittag–Leffler Type Poisson Distribution.

This article is structured as follows:

- The previous methods for image enhancement.
- Definitions related to the proposed algorithm.
- Mathematical approach of the suggested algorithm.
- Experimental analysis and Conclusion.

## Related work

Different techniques, such as blur reduction and denoising, are used to improve images^1^. In order to produce images that are more appropriate than the original images, the majority of image enhancement algorithms rely on spatial processes that are executed on image pixels. Modifying the image histogram is the idea behind image improvement techniques that is used most frequently^2^. Due to its simplicity and adaptability in various situations, this concept is often used. However, when image intensities are normalised, images improved utilising this technique could experience a washed-out look. A brand-new technique based on Joint Histogram Equalisation (JHE) was suggested to address this problem. The goal is to increase visual contrast by utilising neighbouring pixels’ information^3^. Guo et al.^4^, optimised the lighting component iteratively starting with the image’s brightness as the initial illumination component. Utilizing this method results in greater image enhancement with appealing visuals. Hasikin and Mat Isa^5^ suggested an additional fuzzy set theory-based method for improving images. Using this method, improved image quality was attained with little processing time. In their research, the authors suggested a contrast factor that is based on variations in the gray-level values of nearby image pixels. This method was successful in enhancing the image’s quality while retaining its details. Roy et al.^6^ suggested a brand-new Laplacian-based approach for improving fractional calculus enhancement algorithm. The technique was suggested to clear the generated Laplacian noise in text included within video frames. Fu et al.^7^ proposed the fusion-based image improvement approach for images with inadequate illumination by using several techniques to change the image illumination. This strategy successfully increased the illumination of images. Zhang et al.^8^ also suggested a dual illumination estimation-based method for automatic image exposure correction. The multi-exposure image fusion approach is used in this method to transform an input image containing both underexposed and overexposed portions into a well-exposed image overall. Also, Ibrahim et al. ^9^ proposed a model which is based on a class of fractional order heat equations using a hybrid fractional integral-differential operator, which has the potential to improve the image to some extent.

Chen et al.^10^ developed an infrared finger vein picture contrast enhancement based on fuzzy approach for identify recognition. For the enhancement of the acquired infrared image, the fuzzy set theory is employed to address the issues of low contrast, blurring, and speckle noise. The vein pattern feature extract and recognition are used after the gray augmentation. Kumar and Bhandari^11^ proposed a fuzzy c-means clustering method for image enhancement which enhances the perceptually invisible image along with preserving its color and naturalness. In this method, the image pixels are grouped into different clusters and are assigned membership values to those clusters. Based on this membership value, its intensity level is modified in the spatial domain. Yang et al.^12^ proposed a fuzzy c-means clustering with a cooperation center(FCM-co) for image enhancement. Using the FCM-co, the authors divided the image pixels into different clusters and marked membership values to those clusters, modified the membership values and calculated the new pixel gray levels. More recently, Priya and Sruthakeerthi^13^ studied the texture analysis in Image processing using the initial coefficient bounds for a bi-univalent class $C_\Sigma (\lambda ,t,\nu )$ subordinate to Horadam polynomials.

The results discussed above investigated various methods for improving images. The overall appearance is greatly enhanced by these techniques. The main idea of this article is to propose an image enhancement algorithm which uses the convolution of coefficient bounds obtained for a subclass of Sakaguchi kind functions with the image pixels using a $3 \times 3$ mask window. The proposed algorithm yields sufficiently good outcome.

## A set of definitions

Let $\mathcal {A}$ denote the family of analytic functions in the open unit disk $\mathbb {U}=\{\zeta \in \mathbb {C}:|\zeta |<1\}$. These functions *f* are normalized under the conditions $f(0) = 0$ and $f^{\prime }(0)=1$ in $\mathbb {U}$ and are given by,

1 $$\begin{aligned} f(\zeta )=\zeta +\sum _{n=2}^{\infty } o_{n}\zeta ^{n} \end{aligned}$$

and let $\mathcal {S}$ be the subclass of $\mathcal {A}$ consisting of the univalent functions.

Porwal and Dixit^14^ defined the Mittag-Leffler type Poisson distribution as,

$$\begin{aligned} \mathbb {Y}(\lambda ,\delta ,\vartheta )(\zeta ) = \zeta +\sum _{n=2}^{\infty } \frac{\lambda ^{n-1}}{\Gamma (\delta (n-1)+\vartheta )E_{\delta ,\vartheta }(\lambda )} \ \zeta ^n, \end{aligned}$$

where $\mathbb {Y}(\lambda ,\delta ,\vartheta )(\zeta )$ belong to the class $\mathcal {S}$ and,

$$\begin{aligned} \mathbb {Y}(\lambda ,\delta ,\vartheta )(\zeta )(0) = 0 \ \ \text {and} \ \ \mathbb {Y}^{\prime }(\lambda ,\delta ,\vartheta )(\zeta )(0) = 1. \end{aligned}$$

Wiman, Agrawal and others^15,–21^ defined the probability mass distribution of the the Mittag-Leffler type Poisson distribution as,

$$\begin{aligned} \mathbb {P}(\lambda ,\delta ,\vartheta )(\zeta ) = \frac{\lambda ^n}{E_{\delta ,\vartheta }(\lambda )\Gamma (\delta n+\vartheta )}, \ \ n = 0,1,2,..., \end{aligned}$$

where

$$\begin{aligned} E_{\delta ,\vartheta }(\lambda ) = \sum _{k=2}^{\infty } \frac{\lambda ^k}{\Gamma (\vartheta +\delta n)}, \ \ \delta,\vartheta,\lambda \in \mathbb{C}. \end{aligned}$$

Recently, Alessa et al.^22^ studied the Mittag-Leffler type Poisson distribution using convolution operator as,

$$\begin{aligned} \Upsilon (\lambda ,\delta ,\vartheta )f(\zeta ) = \mathbb {Y}(\lambda ,\delta ,\vartheta )(\zeta ) *f(\zeta ) = \zeta +\sum _{n=2}^{\infty } \varrho _\lambda ^n(\delta ,\vartheta ) \ o_n \ \zeta ^n. \end{aligned}$$

where

$$\begin{aligned} \varrho _\lambda ^n(\delta ,\vartheta ) = \frac{\lambda ^{n-1}}{\Gamma (\delta (n-1)+\vartheta )E_{\delta ,\vartheta }(\lambda )}. \end{aligned}$$

Following is the definition for a subclass of Sakaguchi kind functions defined using the Mittag-Leffler type Poisson distribution:

### Definition 1

A function $f \in \mathcal {A}$ is said to be in the class $p-\Upsilon \mathcal {S}^*(t,\delta ,\vartheta )$ if it satisfies the condition

$$\begin{aligned} \Re \left[ \frac{(1-t)\zeta (\Upsilon (\lambda ,\delta ,\vartheta )f(\zeta ))^\prime }{\Upsilon (\lambda ,\delta ,\vartheta ) f(\zeta )- \Upsilon (\lambda ,\delta ,\vartheta ) f(t\zeta )}-1\right] \ge p\left| \frac{(1-t)\zeta (\Upsilon (\lambda ,\delta ,\vartheta )f(\zeta ))^\prime }{\Upsilon (\lambda ,\delta ,\vartheta ) f(\zeta )- \Upsilon (\lambda ,\delta ,\vartheta ) f(t\zeta )}-1\right| . \end{aligned}$$

where $$|t|\le 1,t\ne 1$$.

The following theorem gives the generalized coefficient bound $|o_n|$ for the subclass $p-\Upsilon \mathcal {S}^*(t,\delta ,\mu )$.

### Theorem 1

A function $f \in \mathcal {A}$ of the form (1) is in the class $p-\Upsilon \mathcal {S}^*(t,\delta ,\vartheta )$, then

2 $$\begin{aligned} |o_n| \le \frac{1}{\{(p+1)|(1+t+...+t^{n-1})-n|+|1+t+...+t^{n-1}|\}\Upsilon _\lambda ^n(\delta ,\vartheta )}. \end{aligned}$$

The above result is sharp for the function $g(\zeta )$ given by,

$$\begin{aligned} g(\zeta ) = \zeta + \sum _{n=2}^{\infty }\frac{1}{\{(p+1)|(1+t+...+t^{n-1})-n|+|1+t+...+t^{n-1}|\}\Upsilon _\lambda ^n(\delta ,\vartheta )}\zeta ^n. \end{aligned}$$

By using the initial values obtained from $o_n$ in (2), a $3 \times 3$ mask window is constructed to process the given set of images.

Image Quality Assessment (IQA) is regarded as a characteristic property of an image which measures the degradation of perceived images. Degradation is frequently estimated in comparison to an ideal image. Technical descriptions of image quality are possible, and it is also possible to evaluate it using objectives that highlight variations from ideal or perfect models. It also has to do with how someone could interpret or anticipate an image.

In order to compare the various outcomes of our experiments when working with computer vision challenges, we must select a strategy for gauging the similarity between two images. There are numerous methods and metrics that can be used to evaluate the quality of images. The measuring techniques that are frequently employed in the evaluation of image quality are Peak Signal to Noise Ratio (PSNR), Structural Similarity Index Measure (SSIM), Mean Squared Error (MSE), Root Mean Squared Error (RMSE), Pearson Correlation Coefficient (PCC) and Mean Absolute Error (MAE).

### Definition 2

The PSNR is used to determine the ratio between the maximum signal strength and the power of the noise that distorts the signal’s representation. The decibel form of this ratio between the two images is computed. The PSNR formula is given by,

$$\begin{aligned} \text {PSNR} = 10 \ log_{10} \bigg ( \frac{S^2}{\text {MSE}}\bigg ), \end{aligned}$$

and

$$\begin{aligned} \text {MSE} = \frac{1}{\text{MN}}\sum_{m=1}^\text{M} \sum_{n=1}^\text{N} [I_1(m,n)-I_2(m,n)]^2. \end{aligned}$$

where MSE is the Mean Squared Error which measures the average squared difference between the estimated values and the actual values pixel by pixel, M and N are the number of rows and columns in the images and *S* is the maximum fluctuation in the input image.

### Definition 3

RMSE, Root Mean Squared Error is given by,

$$\begin{aligned} \text {RMSE} = \sqrt{\text {MSE}}. \end{aligned}$$

A smaller RMSE value, like MSE, denotes a closer match or similarity between the images, whereas a bigger RMSE value denotes a greater dissimilarity or inaccuracy.

### Definition 4

The SSIM index is the measure of the similarity between two images.

$$\begin{aligned} \text {SSIM} = \frac{(2\xi _a\xi _b+c_1)(2\varsigma _{ab}+c_2)}{(\xi _a^2+\xi _b^2+c_1)(\varsigma _a^2+\varsigma _b^2+c_2)}, \end{aligned}$$

where $\xi _a$ and $\xi _b$ are the pixel mean of the image windows *a* and *b*, $\varsigma _a^2$ and $\varsigma _b^2$ are the variance of *a* and *b*, $\varsigma _{ab}$ is the covariance of *a* and *b*, $c_1=(r_1\mathcal {L})^2$ and $c_2=(r_2\mathcal {L})^2$ are two variables to stabilize the division, $\mathcal {L}$ is the dynamic range of the pixel values and $r_1=0.01$, $r_2=0.03$ are set by default.

### Definition 5

Pearson Correlation Coefficient (PCC) is used for comparing two images for image registration, disparity measurement, etc.

$$\begin{aligned} r = \frac{\sum_i (x_i-x_m)(y_i-y_m)}{\sqrt{\sum_i (x_i-x_m)^2}\sqrt{\sum_i (y_i-y_m)^2}}, \end{aligned}$$

where $x_i$ and $y_i$ denote the intensity of $i^{th}$ pixel in the input and output image, $x_m$ and $y_m$ denote the mean intensity of the input and output image. The PCC threshold has value 1 if the two images are identical, 0 if they are completely uncorrelated, and -1 if they are completely anti-correlated, for example, if one image is the negative of the other.

### Definition 6

The Mean Absolute Error (MAE) is used to measure the average absolute difference in pixel values between two images. A lower MAE indicates that the distorted image is closer in quality to the original image.

$$\begin{aligned} \text {MAE} = \frac{1}{n} \sum |a_i-b_i|, \end{aligned}$$

where $a_i$ and $b_i$ are the pixel values at the $i^{th}$ position of the input and output image respectively and n is the total number of pixels in the images.

## Proposed work

In this section, we present a mathematical work based on the coefficients obtained for the class given by $p-\Upsilon \mathcal {S}^*(t,\delta ,\mu )$.

The coefficients obtained using $o_n$ are used to enhance the images. The processed image can be denoted by $I_E(m,n)$ which can be obtained by the convolution,

$$\begin{aligned} I_E(m,n) = \mathcal {M} *I_O(m,n), \end{aligned}$$

where $I_O(m,n)$ is the original image, $\mathcal {M}$ is the $3 \times 3$ mask window and $*$ is the convolution operation. The mask window is represented using the coefficients $o_1, o_2$ and $o_3$ as follows:

$$\begin{aligned} o_{0^\circ } = \begin{array}{|c|c|c|} \hline 0 &{} 0 &{} 0 \\ \hline o_1 &{} o_2 &{} o_3 \\ \hline 0 &{} 0 &{} 0 \\ \hline \end{array} \quad o_{90^\circ } = \begin{array}{|c|c|c|} \hline 0 &{} o_3 &{} 0 \\ \hline 0 &{} o_2 &{} 0 \\ \hline 0 &{} o_1 &{} 0 \\ \hline \end{array} \quad o_{45^\circ } = \begin{array}{|c|c|c|} \hline 0 &{} 0 &{} o_3 \\ \hline 0 &{} o_2 &{} 0 \\ \hline o_1 &{} 0 &{} 0 \\ \hline \end{array} \quad o_{135^\circ } = \begin{array}{|c|c|c|} \hline o_3 &{} 0 &{} 0 \\ \hline 0 &{} o_2 &{} 0 \\ \hline 0 &{} 0 &{} o_1 \\ \hline \end{array} \end{aligned}$$

The initial coefficients for the class $p-\Upsilon \mathcal {S}^*(t,\delta ,\vartheta )$ with $t=-0.5, \delta =1, \lambda =0.01,$ and $p=4999$ are given by,

$$\begin{aligned} o_1 &= 1, \\ o_2 &= \frac{\Gamma (1+\vartheta )\sum _{k=2}^{\infty }\frac{0.01^k}{\Gamma (\vartheta +k)}}{75.005}, \\ o_3 &= \frac{\Gamma (2+\vartheta )\sum _{k=2}^{\infty }\frac{0.01^k}{\Gamma (\vartheta +k)}}{1.125075}. \end{aligned}$$

The pixel values of the proposed mask are calculated by sliding the mask window over the grayscale pixel values of the original image, generally starting with the top-left corner of the image and moving through all the pixels until the fractional mask fits completely on the image.

The proposed mask window for image enhancement can be obtained from the following algorithm:

Step 1: Convert the RGB images to Grayscale images.
Step 2: Set the proposed mask window to a $3 \times 3$ pixel size.
Step 3: Fix the range of $\vartheta$ as $0< \vartheta < 1$.
Step 4: Apply the convolution of fractional mask in four directions over the original image.
Step 5: Calculate the quality metrics for the enhanced image.

**Figure 1:**
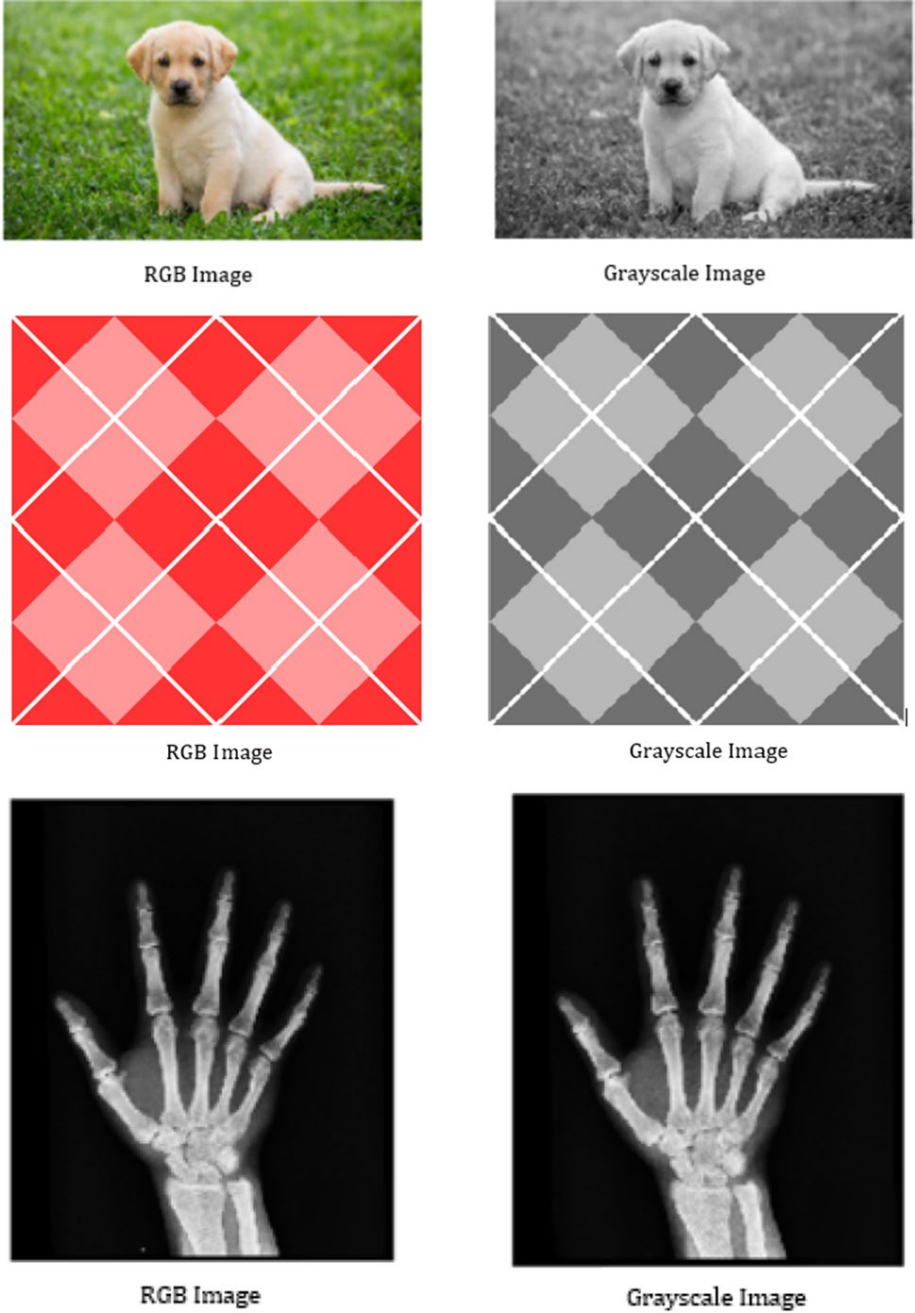
Conversion of RGB images “DOG”, “ARGYLE” and “X-RAY” to Grayscale images.

The above algorithm is initially applied to various types of images with different pixel values to check its functioning. For this purpose, we used the RGB images “DOG”, “ARGYLE” and “X-RAY” which are converted to Grayscale images given in Fig. 1.

**Figure 2:**
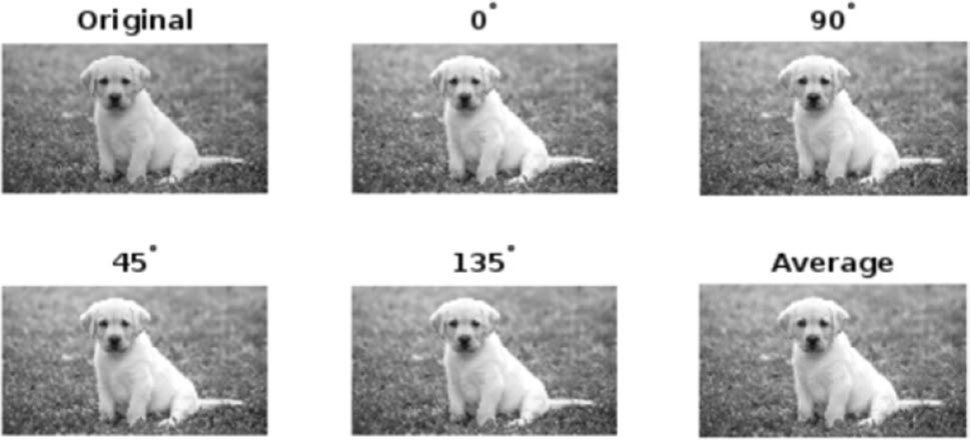
Enhancement of “DOG” at various mask angles.

**Figure 3:**
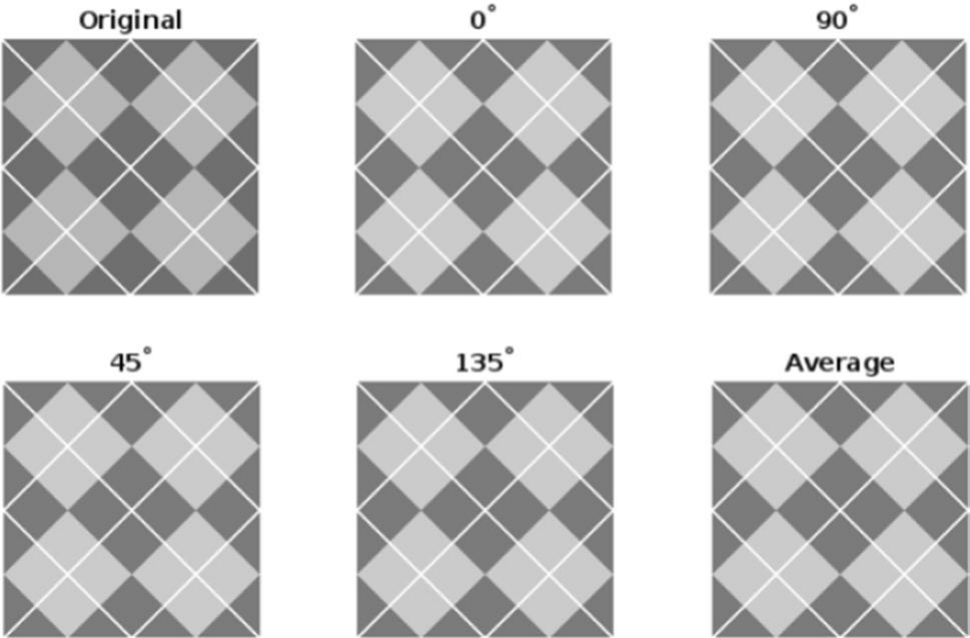
Enhancement of “ARGYLE” at various mask angles.

**Figure 4:**
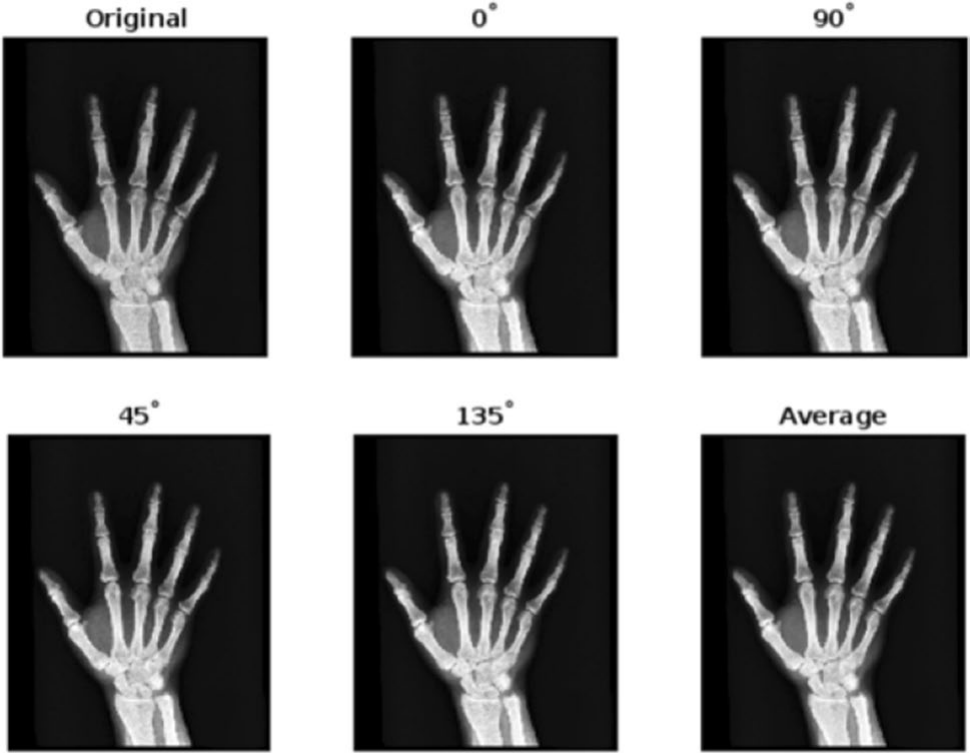
Enhancement of “X-RAY” at various mask angles.

## Experimental results and analysis

The effectiveness of the suggested algorithm is shown in this section. Performance evaluations are carried out using MATLAB online. PSNR, SSIM, MSE, RMSE, PCC and MAE, which are frequently used in literature to assess the quality of a processed image, is taken into account to confirm the image’s quality.

**Figure 5:**
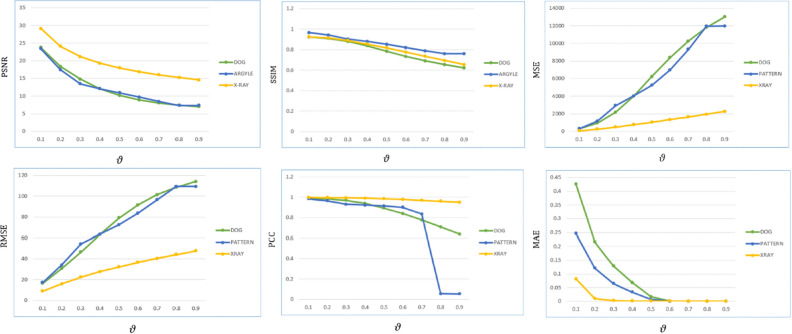
Graphical Representation of Tables 1, 2, 3.

**Figure 6:**
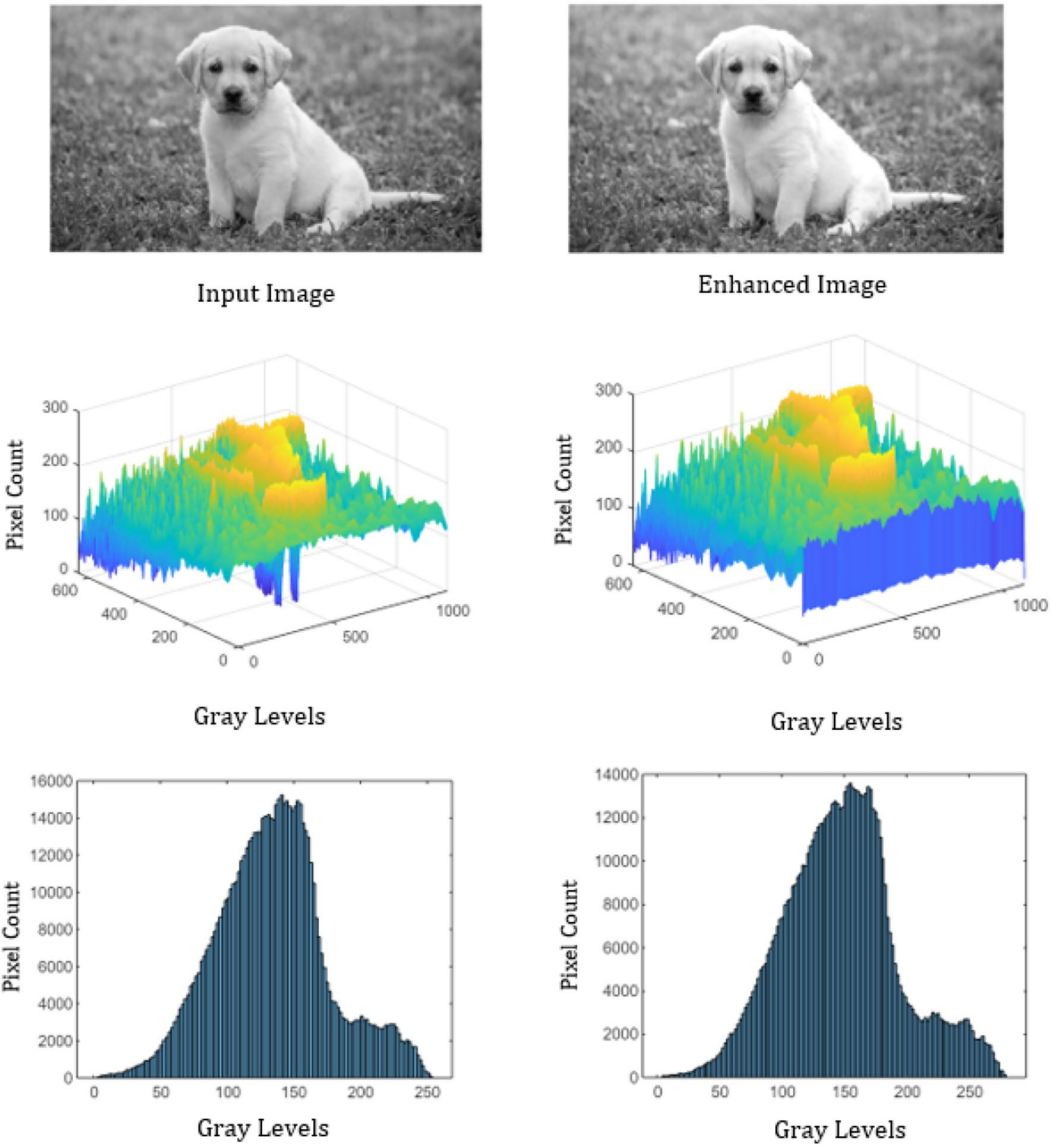
Experimental results for the image “DOG”.

**Figure 7:**
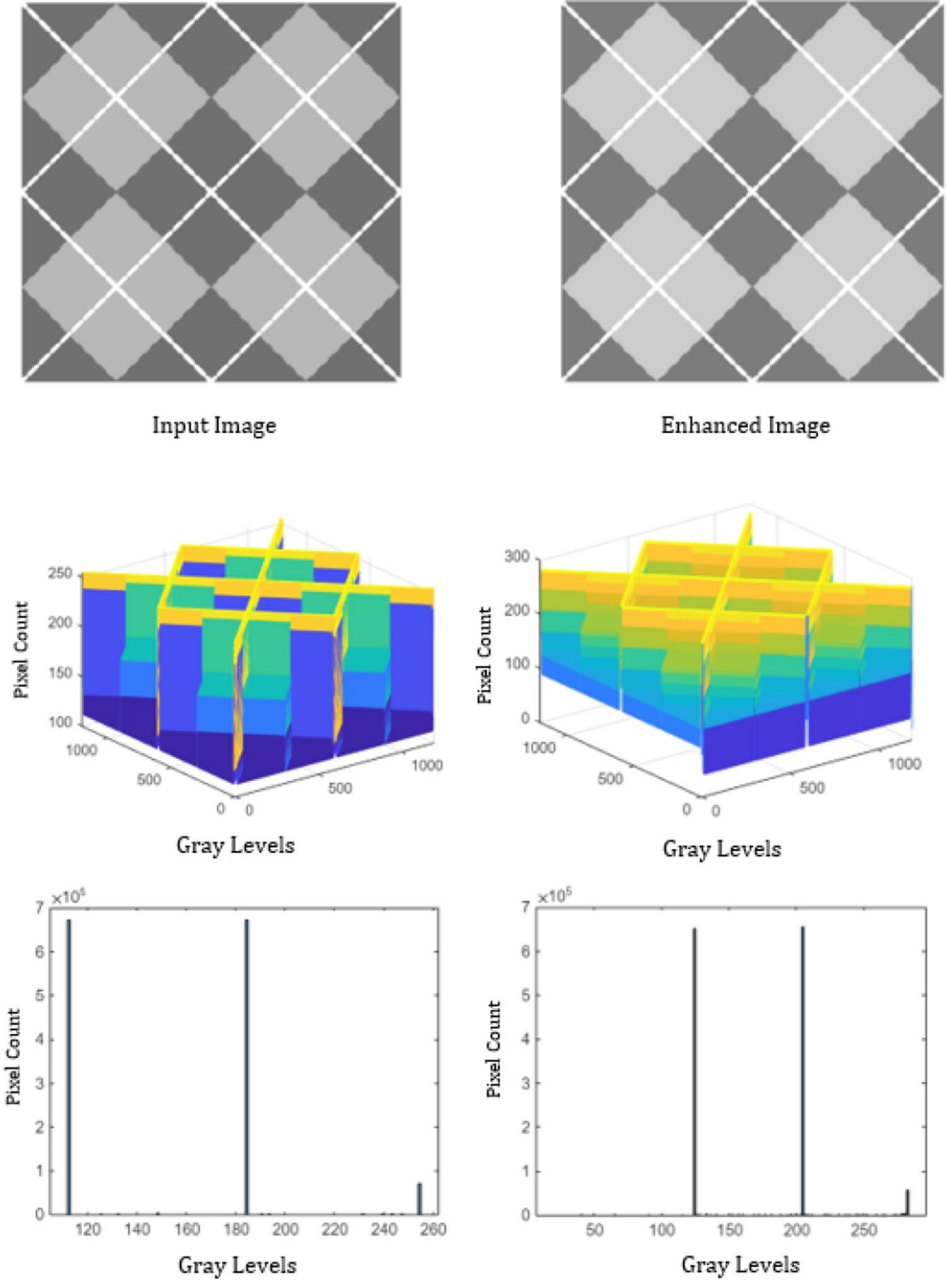
Experimental results for the image “ARGYLE”.

**Figure 8:**
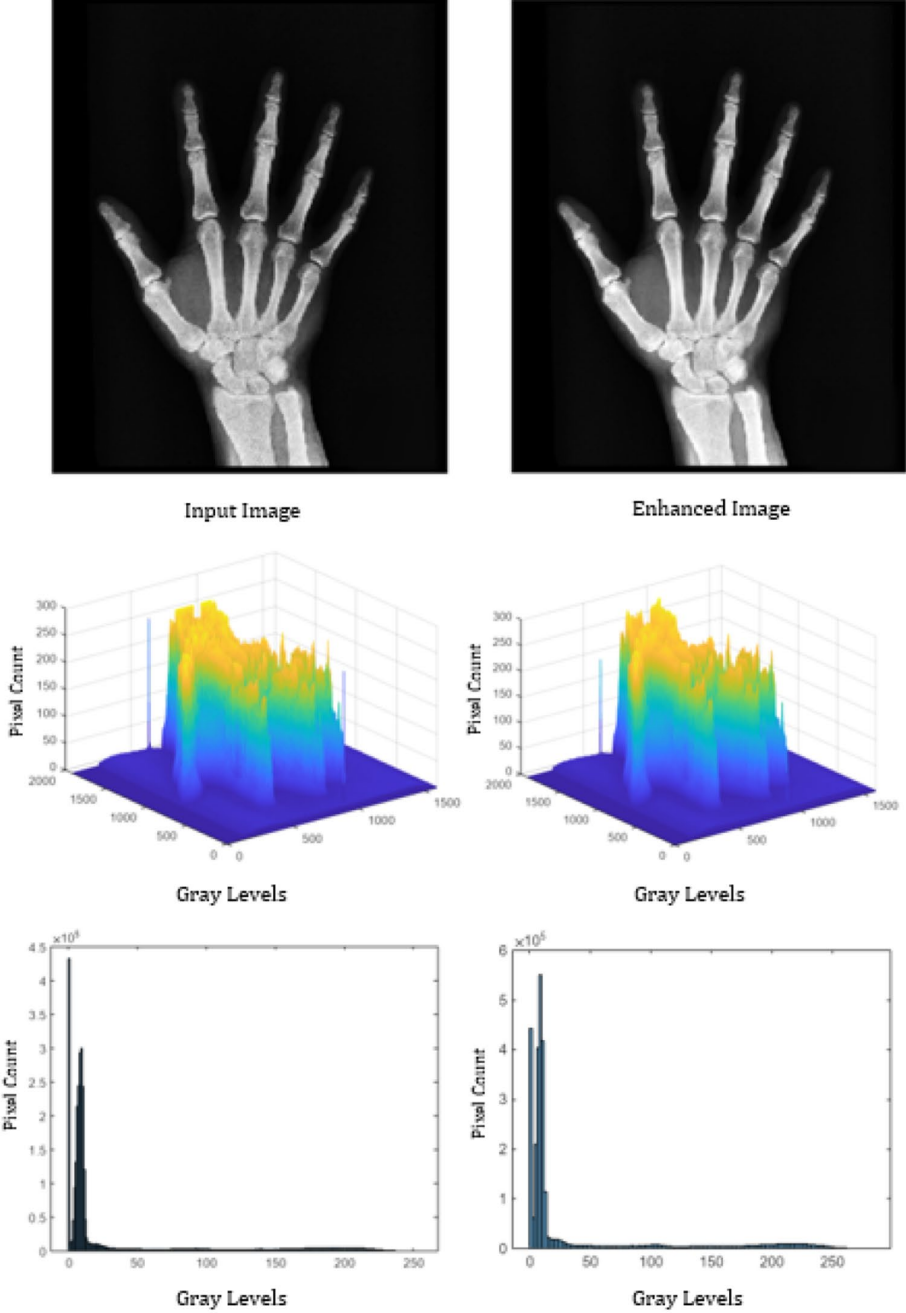
Experimental results for the image “X-RAY”.

To analyze the performance, the proposed algorithm is tested on images: “DOG” of size $1120\times 640$, “ARGYLE” pattern of size $1200\times 1200$ and “X-RAY” of size $1597\times 1920$. Figures 2, 3 and 4 gives the enhancement of the images “DOG”, “ARGYLE” and “X-RAY” for different angles $0^\circ$, $90^\circ$, $45^\circ$, $135^\circ$ and the average of all the angles at $\vartheta =0.1$. Tables 1, 2, 3 gives the observation of the quality metrics for the images at different values of $\vartheta$ and it is noted that at $\vartheta =0.1$, the quality metrics are optimum for all the images. The graphical representation of Tables 1, 2, 3 is given in Figure 5. Figures 6, 7 and 8 illustrates the input image and enhanced images of “DOG”, “ARGYLE” and “X-RAY” along with the representations mesh and histogram respectively. At $\vartheta =0.1$, it is observed that the quality metrics are higher for the proposed algorithm.

**Table 1:** Observation of Quality metrics for “DOG” at different values of $\vartheta$..

$$t=-0.5, \delta =1, \lambda =0.01, p=4999$$						
$\vartheta$	PSNR	SSIM	MSE	RMSE	PCC	MAE
0.1	23.7637	0.9236	273.3444	16.5331	0.9857	0.4258
0.2	18.3782	0.9098	944.6358	30.7349	0.9825	0.2162
0.3	14.7748	0.8799	$2.17 \times 10^3$	46.5373	0.9684	0.1288
0.4	12.1043	0.8370	$4.01\times 10^3$	63.2888	0.9384	0.0688
0.5	10.1811	0.7853	$6.24\times 10^3$	78.9743	0.8937	0.0166
0.6	8.8944	0.7339	$8.39\times 10^3$	91.5841	0.8394	$4.09\times 10^{-4}$
0.7	8.0151	0.6889	$1.03\times 10^4$	101.3411	0.7767	$1.77\times 10^{-4}$
0.8	7.4061	0.6529	$1.18\times 10^4$	108.7013	0.7085	$9.49\times 10^{-5}$
0.9	6.9842	0.6228	$1.30\times 10^4$	114.1119	0.6381	$6.70\times 10^{-5}$

**Table 2:** Observation of Quality metrics for “ARGYLE” at different values of $\vartheta$..

$$t=-0.5, \delta =1, \lambda =0.01, p=4999$$						
$\vartheta$	PSNR	SSIM	MSE	RMSE	PCC	MAE
0.1	23.4281	0.9660	295.3038	17.1844	0.9836	0.2458
0.2	17.4551	0.9426	$1.17 \times 10^3$	34.1809	0.9649	0.1214
0.3	13.4381	0.9036	$2.95\times 10^3$	54.2794	0.9314	0.0647
0.4	12.0849	0.8798	$4.02\times 10^3$	63.4302	0.9225	0.0337
0.5	10.9303	0.8524	$5.25\times 10^3$	72.4481	0.9161	0.0063
0.6	9.6949	0.8203	$6.98\times 10^3$	83.5214	0.9006	$2.07\times 10^{-4}$
0.7	8.4440	0.7882	$9.30\times 10^3$	96.4588	0.8358	$1.51\times 10^{-4}$
0.8	7.3551	0.7598	$1.20\times 10^4$	109.3414	0.0557	$9.03\times 10^{-5}$
0.9	7.3526	0.7604	$1.20\times 10^4$	109.3733	0.0546	$6.04\times 10^{-5}$

**Table 3:** Observation of Quality metrics for “X-RAY” at different values of $\vartheta$..

$$t=-0.5, \delta =1, \lambda =0.01, p=4999$$						
$\vartheta$	PSNR	SSIM	MSE	RMSE	PCC	MAE
0.1	29.1316	0.9244	79.4181	8.9117	0.9974	0.0815
0.2	24.1134	0.9145	252.1953	15.8807	0.9972	0.0095
0.3	21.1487	0.8891	499.1213	22.341	0.9951	0.0025
0.4	19.2919	0.8545	765.405	27.666	0.9909	0.0012
0.5	17.9495	0.8182	$1.04\times 10^3$	32.2897	0.9849	$9.05\times 10^{-4}$
0.6	16.8632	0.7768	$1.34\times 10^3$	36.5916	0.9776	$8.06\times 10^{-4}$
0.7	15.9846	0.7359	$1.64\times 10^3$	40.4866	0.9695	$4.71\times 10^{-4}$
0.8	15.2312	0.6938	$1.95\times 10^3$	44.1551	0.9607	$4.08\times 10^{-4}$
0.9	14.5545	0.6541	$2.28\times 10^3$	47.7328	0.9513	$3.54\times 10^{-4}$

Also, the algorithm is tested on 3 different data sets to check its robustness and stability including “DAISY”, “MEDICAL” and “MISCELLANEOUS” data sets. The “DAISY” data set consists of 764 images with size around $320 \times 240$ pixels and ’.jpg’ extension. The “MEDICAL” data set consists of 200 images with size around $1514 \times 2044$ pixels and ’.png’ extension. The “MISCELLANEOUS” data set consists of 44 images with size around $512 \times 512$ pixels and ’.tiff’ extension. The data sets are selected with different properties taken into account like number of images, size and extensions so as to validate the working of the proposed algorithm.

**Table 4:** Average values of the quality metrics for the given data sets..

Data set	PSNR	SSIM	MSE	RMSE	PCC	MAE
DAISY	22.5662	0.8239	425.9394	19.7698	0.9590	1.9359
MEDICAL	31.2983	0.9614	61.886	7.4082	0.9593	0.1313
MISCELLANEOUS	23.4508	0.7523	337.2533	18.2404	0.9418	1.1570

**Figure 9:**
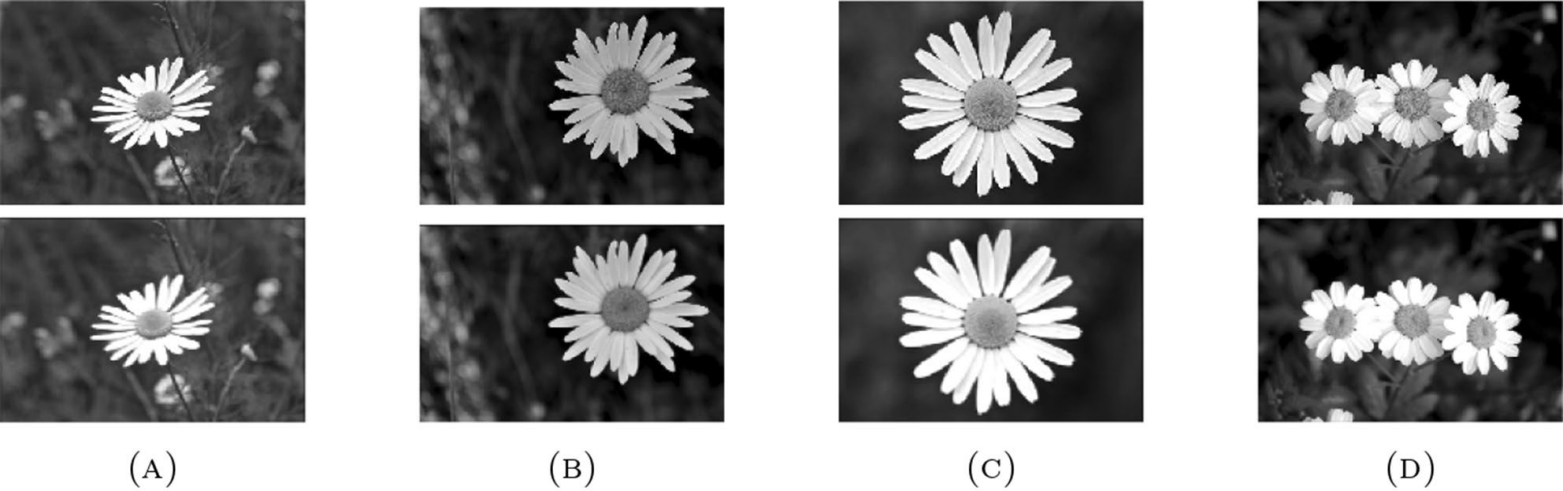
Enhancement of sample images from “DAISY”.

**Figure 10:**
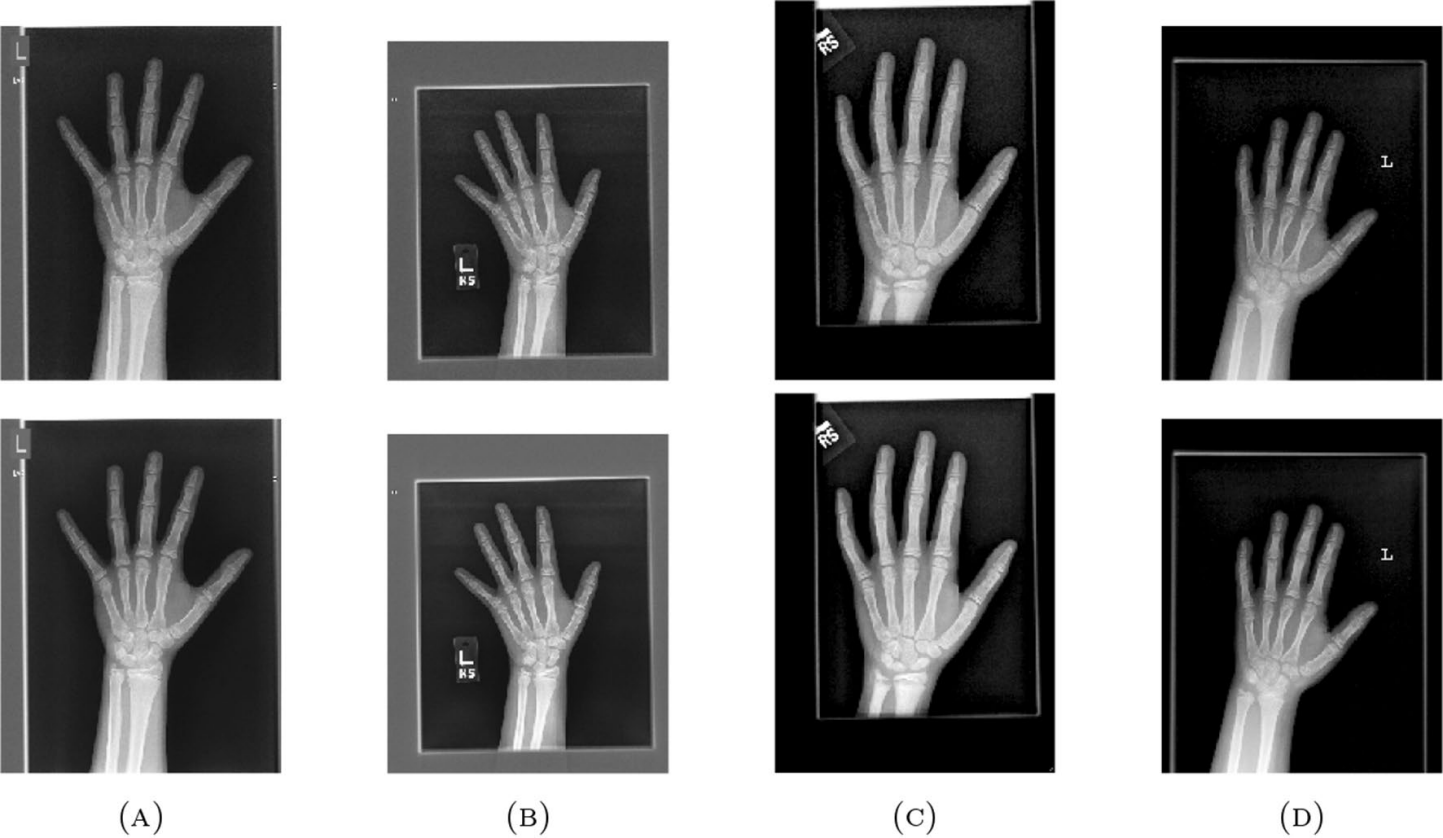
Enhancement of sample images from “MEDICAL”.

**Figure 11:**
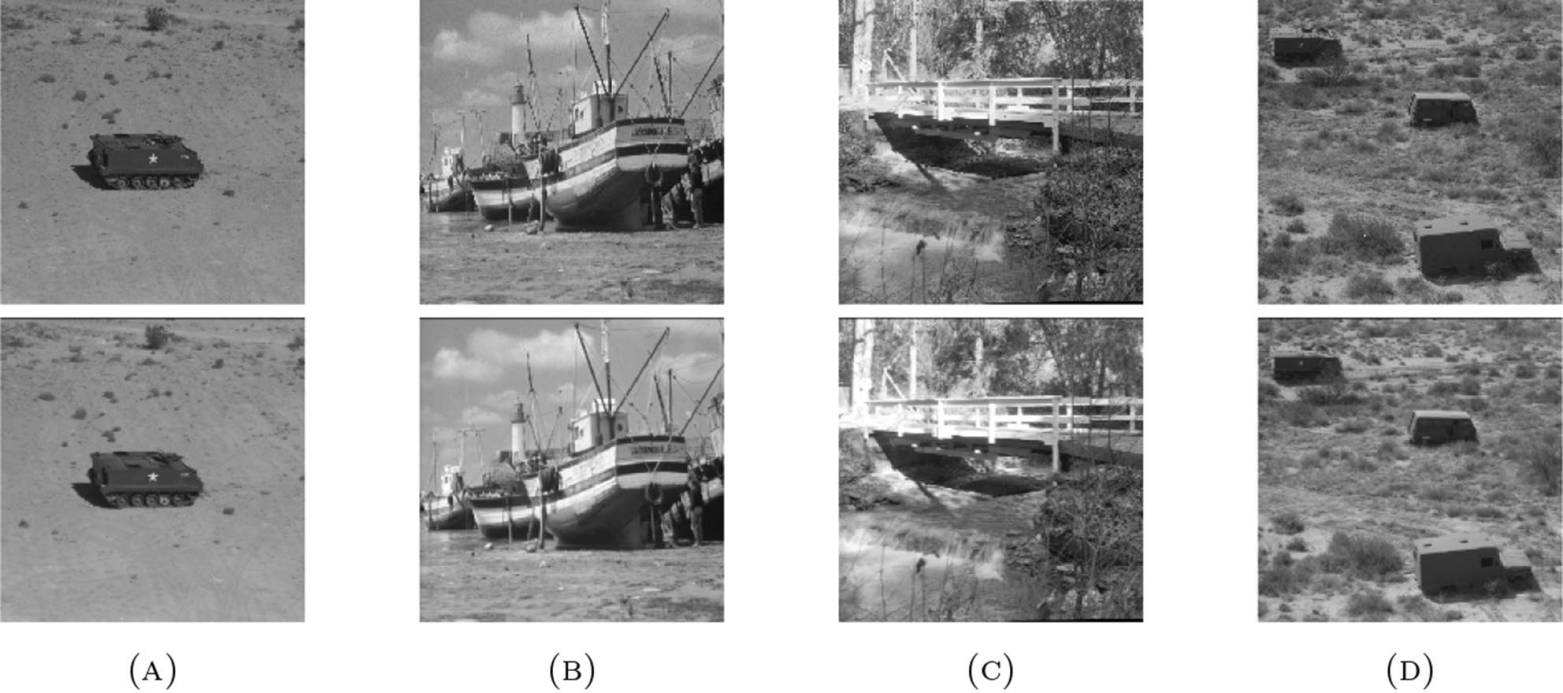
Enhancement of sample images from “MISCELLANEOUS”.

The values of the quality metrics PSNR, SSIM, MSE, RMSE, PCC and MAE for the average of all images in each data set are listed in Table 4. The enhancement of the images is optimum at $\vartheta =0.1$. Figures 9, 10 and 11 shows the enhancement of sample images from the given data sets using the proposed algorithm.

In conclusion, the proposed algorithm is capable of delivering optimal results for images of any size and diverse file formats, including PNG, JPEG, BMP, TIFF, GIF, HEIC, DICOM, and more. As illustrated in Fig. 5, the quality metrics achieved for the images demonstrate a high level of satisfaction. A key benefit of this algorithm lies in its ability to be applied to images with a wide range of extensions and substantial pixel values.

## Conclusion

The key concept of this paper is to propose a novel image enhancement algorithm which is simple and easy to access. The proposed algorithm is based on a new convolution technique using the coefficient bounds obtained for the class $p-\Upsilon \mathcal {S}^*(t,\delta ,\mu )$ of analytic functions applied on a $3\times 3$ mask window. It is observed that by decreasing the parameter value $\vartheta$, the enhancement of the images is improved and the quality metrics are ideal at $\vartheta =0.1$. Since development in computer technology and imaging is increasing rapidly, our work can be applied in various areas of image processing including image restoration, sharpening, edge detection and gamma correction.

## Data Availability

The following links are given for the images and data sets used in this study. Source file of “DOG”. Source file of “ARGYLE”. Source file of “X-RAY”. Source file of Data set 1—“DAISY”. Source file of Data set 2—“MEDICAL”. Source file of Data set 3—“MISCELLANEOUS”.
